# Efficient production of CRISPR/Cas9 gene knockouts in the male/female nematode *Caenorhabditis nigoni*

**DOI:** 10.17912/micropub.biology.000968

**Published:** 2023-09-06

**Authors:** Jonathan P Harbin, Ronald E Ellis

**Affiliations:** 1 Molecular Biology, Rowan-Virtua SOM, Stratford, New Jersey, United States

## Abstract

Although nematode genetics was founded on the use of hermaphrodite genetics for studying animal development and behavior, there is a growing need to extend this work to male/female species. One of the most promising species is *C. nigoni,* because it is so closely related to the model hermaphroditic *C. briggsae.* We present methods for using CRISPR/Cas9 gene editing to create mutations, and techniques for balancing, maintaining and studying these mutations.

**Figure 1. Workflow to produce and identify Cas9-induced gene knockouts in C. nigoni:**
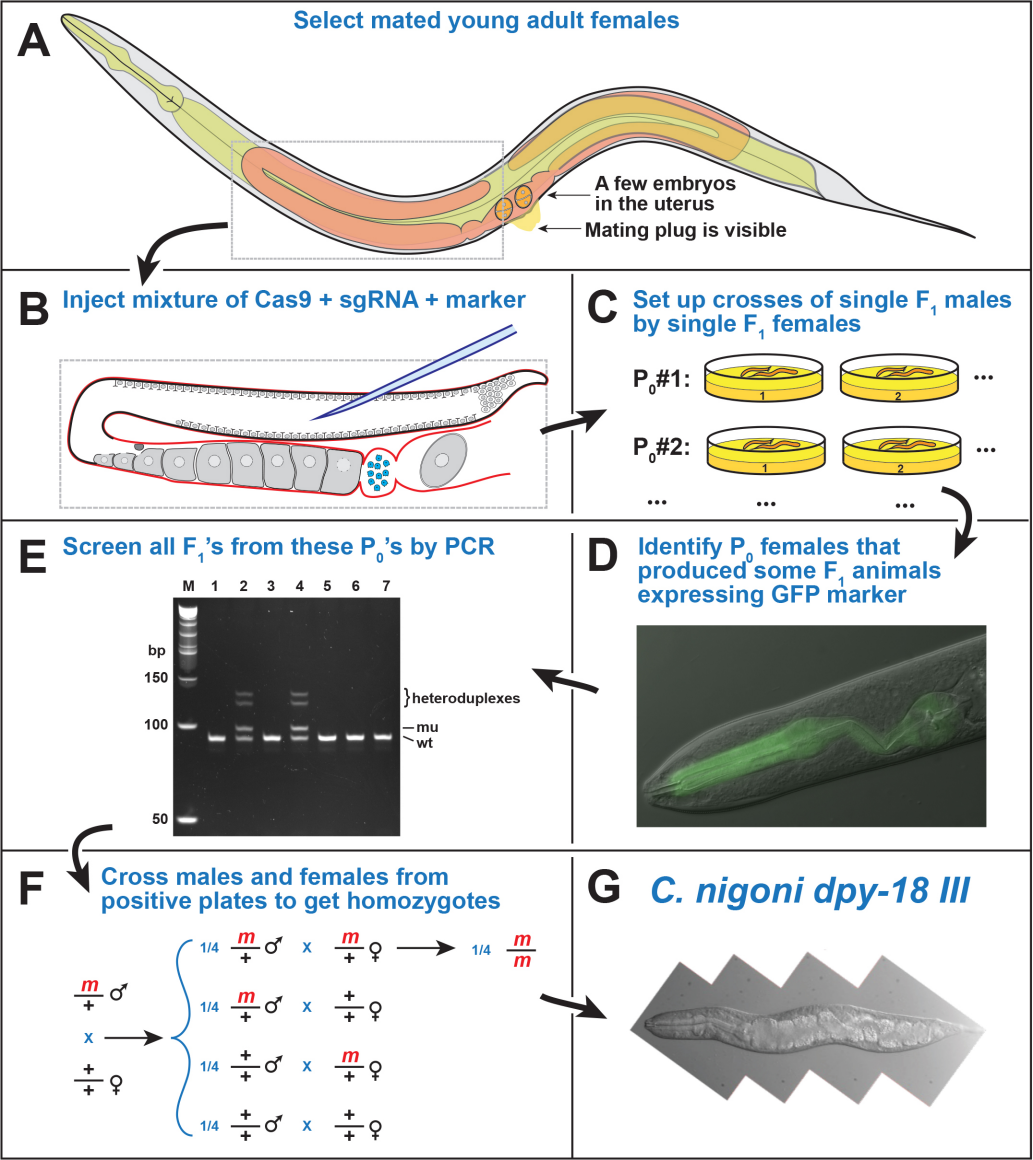
This workflow represents the steps needed to produce directed genome edits in *C. nigoni* . (A) Select young gravid females for microinjection, to ensure large and reliable F _1_ broods. Ideally, there should only be a few embryos in the uterus and a visible mating plug (previously deposited by a male over the vulva). (B) Use standard techniques for microinjection of *Caenorhabditis* nematodes (Berkowitz *et al.* , 2008). The injection mixture should contain Cas9/sgRNA RNP complexes (Paix *et al.* , 2014), a selection marker (pMyo-2::GFP plasmid) and a repair template, if one is needed. (C) If possible, co-inject an easily detectable selection marker. We use a GFP expression plasmid controlled by the pharyngeal myosin promotor, pMyo-2, and screen the F _1_ progeny with a fluorescence stereo microscope to identify P _0_ females that were successfully injected. (D) Cross pairs of F _1_ males and females to produce F _2_ descendants, then harvest the parents of successful broods for PCR, and screen them for indels. Use as many sibling pairs as possible. Cross surplus F _1_ animals with wildtype animals of the opposite sex. After reproduction, lyse the F _1_ parents to isolate genomic DNA. Amplify DNA from the target site (~100 bp) using standard PCR methods and screen for a mobility shift on 8% acrylamide gels. This approach can be used to detect small insertions or deletions, or precise edits such as a tag or new restriction site. Roughly 2/3 of the indel mutations result in a frameshift, which should produce a premature stop codon. Sequence mutant alleles promptly to avoid expending resources on undesirable mutations. (E) Following identification of a new mutant, use sibling crosses to make homozygotes. Alternatively, use crosses with animals that carry a balancer mutation to establish a stable heterozygous strain. (F) Once homozygotes are identified they can be studied for their unique properties, like this *C. nigoni dpy-18( v484 )* mutant.

## Description

*Caenorhabditis* nematodes provide a powerful system for studying many aspects of animal development and behavior. One reason that *C. elegans* became so widely studied is that the *XX* animals are self-fertile hermaphrodites. Although this trait simplifies genetic analysis, females are far more common among *Caenorhabditis* species and among animals more broadly. However, few studies have analyzed female nematodes from this genus. Instead, the self-fertile species *C. briggsae* is widely used for comparison with *C. elegans* (Gupta *et al.* , 2007). Since both species evolved hermaphrodites independently (reviewed by Ellis & Lin, 2014; Ellis, 2016), they offer two examples of how mating systems can change during evolution.

Because self-fertility simplifies experiments, less work has been done with the non-hermaphroditic species (e.g. Baldi *et al.* , 2009; Yin *et al.* , 2018). Among the male/female species in this group, *C. nigoni* is of particular interest, due to its close relationship to the self-fertile species *C. briggsae* (Woodruff *et al.* , 2010). Studying *C. nigoni* could illuminate the state of their common ancestor, shed light on female traits, and help us understand the origin of self-fertility.

Although *C. nigoni* is not yet practical for forward genetic screens, the CRISPR/Cas9 system makes reverse genetics possible (Lo *et al.* , 2013). Here we present the techniques and approaches we developed for using CRISPR/Cas9 in *C. nigoni .*

These techniques need to address several problems that do not exist when working with hermaphroditic nematodes: (1) Male/female species have a high level of genetic variability (e.g. Dey *et al.* , 2013), which makes them difficult to inbreed to isogenicity. Thus strains retain some genetic diversity. (2) One consequence of this is that most partially inbred strains carry mutations that reduce their mating efficiency and brood size (Chelo *et al.* , 2014). Moreover, some of these mutations could potentially alter the phenotypes of other genes *.* (3) Every step in the gene editing process involves a cross with two parents. (4) Many mutations that are easily maintained in *C. elegans* or *C. briggsae* prevent mating in *C. nigoni ,* which requires the use of balancers. (5) The genome sequence is not complete, so balancer mutations must be identified from a fragmentary genetic map.

Unlike *C. elegans* and *C. briggsae ,* the dioecious species, *C. nigoni ,* is not self-fertile and can only reproduce by mating. As a result, there is always the possibility that critical crosses will fail. Furthermore, if the genotypes of both parents are uncertain, this increases the number of progeny that must be tested to identify key animals. And it necessitates finding both an appropriate male and female at the same time, so they can mate and propagate the mutation.

We use several steps to address these problems. First, we inject mated females with CRISPR/Cas9 solution, so they do not need to mate after injection. These protein/guide RNA injections not only work in every species we have tried, but they also give a high probability of inducing a new mutation in a short time window, after which the injection components break down, making the chance of confusing somatic mutations low. Hence, we prefer them to editing with plasmids.

Second, we set up crosses between pairs of F _1_ progeny to look for new mutations. Thus, either of these F _1_ parents could potentially pass on a new CRISPR allele. Furthermore, if two F _1_ s successfully reproduce, we harvest both parents in a single tube for PCR analysis. Since screening for new CRISPR alleles can involve hundreds of F _1_ animals and PCR reactions, these simple steps significantly lower the time needed for the process.

Third, when studying *C. elegans ,* even lethal or sterile mutations are easily maintained by selecting heterozygotes, which can be identified by the ¼ mutant progeny in their broods. Since this is not possible with diecious animals, we follow all mutations by PCR analysis of the parents, who are harvested after mating and laying eggs. Furthermore, we balance each mutation as soon as possible. Crosses to produce balanced strains usually take considerable effort because so many pairs of F _1_ or F _2_ animals must be allowed to mate, to find the correct combination of parents.

Unfortunately, since *C. nigoni* is a relatively new research model, common reagents and strains carrying morphological markers are rare. Thus, for genetic balancers, we use morphological markers near genes of interest in the genome assembly.

However, these morphological markers will probably have to be generated in parallel with the target gene. We make each mutation and balancer separately and create balanced strains through crosses. (Attempts to generate both mutations from a single injection often resulted in *cis* mutations that were very closely linked).

There are multiple lines of *C. nigoni* available, each derived from different wild isolates and subjected to varying levels of inbreeding. However, all of these laboratory strains are less healthy than *C. briggsae* or *C. elegans* strains *.* We suspect that the variability in phenotype and small broods we observe are due to inbreeding depression and residual heterozygosity.

So far this has not prevented us from isolating homozygous animals for each locus we have targeted, but the mutants are sicker than corresponding mutants in *C. briggsae .* And it remains possible that there might be some loci that cannot be made homozygous in certain *C. nigoni* strains, because of balanced lethal mutations in the region.

To circumvent many of these problems, we use interstrain hybrids (e.g. see Yin *et al.* , 2018). These animals are generated by crossing parents of two different genetic backgrounds that each carry a mutation in the gene of interest. The two *C. nigoni* strains used in our work are JU1422 (Woodruff *et al.* , 2010) and CP168 (E. Haag and E. Schwarz, pers. comm.).

Generally, the JU1422 / CP168 interstrain hybrids grow and reproduce more vigorously, which results in larger broods and increases accuracy and efficiency when scoring mutant phenotypes. Thus, using interstrain hybrids reduces genetic background effects. In addition, small nucleotide polymorphisms between the two strains can be used to follow maternal and paternal chromosomes in crosses. This has the added benefit of being a convenient method to karyotype the *X* chromosome and aids in the generation and characterization of double mutants.

Making these alleles requires good information about the genomes of each strain. The first *C. nigoni* genome sequenced was from the inbred strain JU1422 (Yin *et al.* , 2018), and is easily available through WormBase (Davis *et al.* , 2022). We also use the new inbred strain CP168 , which was derived from a different wild isolate; this strain and its genome assembly were recently shared with us (E. Haag and E. Schwarz, pers. comm.).

These draft genomes are complete enough for reverse genetic approaches, but they still have many gaps, and each chromosome is represented by many separate scaffolds. The BUSCO completeness scores for the JU1422 and CP168 genome assembly are 98.9% and 98.5%, respectfully. Thus, finding linked pairs of genes to establish balanced strains can be a challenge. In our experience, genes with visible phenotypes (based on studies of their *C. elegans* orthologs) can usually be found near genes of interest on the same scaffold, and are generally close enough that recombination between mutation and balancer is very infrequent. The more complete *C. briggsae* genome can also be used to infer linkage between *C. nigoni* balancers, due to the high synteny between the *C. briggase* and *C. nigoni* genomes (Yin *et al.* , 2018).

Using these methods, we have already made mutations in seven different *C. nigoni* genes across three chromosomes. The accompanying paper by Choi and Villeneuve describes a co-CRISPR method for *C. nigoni* that should speed up this process significantly (Choi and Villeneuve, 2023) . None of the mutants we identified can be maintained as a homozygous stock, but all have been balanced, and homozygotes isolated for study. Future analysis of traits with phenotypes that do not affect mating should be much easier. Furthermore, markers using GFP could also be valuable, just as they have been in *C. briggsae* (Bi *et al.* , 2015).

## Methods

Nematode strains were cultured as described previously (Brenner, 1974) .

We follow protocols for CRISPR/Cas9 microinjection first developed by Paix *et al.* (2015 Suppl. File S1), as refined by Ghanta *et al.* (2021).

## Reagents

The inbred *C. nigoni* strain JU1422 (Woodruff *et al.* , 2010) was provided by the *Caenorhabditis* Genetics Center. The inbred *C. nigoni* strain CP168 , (further inbred from EG5268 ) was a gift from Eric Haag and Erich Schwarz. The *Cni-dpy-18* locus is based on genome assembly: PRJNA384657, Scaffold: CM008511.1, Location: 6615132..6617960.

The *pmyo-2* ::GFP plasmid we use was created from the plasmid PDD04 (obtained from Addgene) which contained a *C. elegans* promoter. We removed the *pmyo-2* promoter region and replaced it with a 500 bp fragment immediately upstream of the *cbr-myo-2* 5’UTR *.* This species-specific promotor region increased pharynx GFP expression for *C. briggsae* and *C. nigoni.*

## References

[R1] Baldi C, Cho S, Ellis RE (2009). Mutations in two independent pathways are sufficient to create hermaphroditic nematodes.. Science.

[R2] Berkowitz LA, Knight AL, Caldwell GA, Caldwell KA (2008). Generation of stable transgenic C. elegans using microinjection.. J Vis Exp.

[R3] Bi Y, Ren X, Yan C, Shao J, Xie D, Zhao Z (2015). A Genome-wide hybrid incompatibility landscape between Caenorhabditis briggsae and C. nigoni.. PLoS Genet.

[R4] Brenner S (1974). The genetics of Caenorhabditis elegans.. Genetics.

[R5] Chelo IM, Carvalho S, Roque M, Proulx SR, Teotónio H (2013). The genetic basis and experimental evolution of inbreeding depression in Caenorhabditis elegans.. Heredity (Edinb).

[R6] Choi C, Villeneuve M. 2023. CRISPR/Cas9 mediated genome editing of Caenorhabditis nigoni using the conserved dpy-10 co-conversion marker. microPublication Biology. 10.17912/micropub.biology.000937 10.17912/micropub.biology.000937 PMC10500344 37720684

[R7] Davis P, Zarowiecki M, Arnaboldi V, Becerra A, Cain S, Chan J, Chen WJ, Cho J, da Veiga Beltrame E, Diamantakis S, Gao S, Grigoriadis D, Grove CA, Harris TW, Kishore R, Le T, Lee RYN, Luypaert M, Müller HM, Nakamura C, Nuin P, Paulini M, Quinton-Tulloch M, Raciti D, Rodgers FH, Russell M, Schindelman G, Singh A, Stickland T, Van Auken K, Wang Q, Williams G, Wright AJ, Yook K, Berriman M, Howe KL, Schedl T, Stein L, Sternberg PW (2022). WormBase in 2022-data, processes, and tools for analyzing Caenorhabditis elegans.. Genetics.

[R8] Dey A, Chan CK, Thomas CG, Cutter AD (2013). Molecular hyperdiversity defines populations of the nematode Caenorhabditis brenneri.. Proc Natl Acad Sci U S A.

[R9] Ellis RE (2016). "The persistence of memory"-Hermaphroditism in nematodes.. Mol Reprod Dev.

[R10] Ellis RE, Lin SY (2014). The evolutionary origins and consequences of self-fertility in nematodes.. F1000Prime Rep.

[R11] Ghanta KS, Ishidate T, Mello CC (2021). Microinjection for precision genome editing in
*Caenorhabditis elegans*
.. STAR Protoc.

[R12] Gupta BP, Johnsen R, Chen N (2007). Genomics and biology of the nematode Caenorhabditis briggsae.. WormBook.

[R13] Lo TW, Pickle CS, Lin S, Ralston EJ, Gurling M, Schartner CM, Bian Q, Doudna JA, Meyer BJ (2013). Precise and heritable genome editing in evolutionarily diverse nematodes using TALENs and CRISPR/Cas9 to engineer insertions and deletions.. Genetics.

[R14] Paix A, Folkmann A, Rasoloson D, Seydoux G (2015). High Efficiency, Homology-Directed Genome Editing in Caenorhabditis elegans Using CRISPR-Cas9 Ribonucleoprotein Complexes.. Genetics.

[R15] Paix A, Wang Y, Smith HE, Lee CY, Calidas D, Lu T, Smith J, Schmidt H, Krause MW, Seydoux G (2014). Scalable and versatile genome editing using linear DNAs with microhomology to Cas9 Sites in Caenorhabditis elegans.. Genetics.

[R16] Woodruff GC, Eke O, Baird SE, Félix MA, Haag ES (2010). Insights into species divergence and the evolution of hermaphroditism from fertile interspecies hybrids of Caenorhabditis nematodes.. Genetics.

[R17] Yin D, Schwarz EM, Thomas CG, Felde RL, Korf IF, Cutter AD, Schartner CM, Ralston EJ, Meyer BJ, Haag ES (2018). Rapid genome shrinkage in a self-fertile nematode reveals sperm competition proteins.. Science.

